# Exploring the Interplay Among a Health-Promoting Lifestyle, Wellbeing, and Sociodemographic Characteristics in Italy: A Cross-Sectional Study

**DOI:** 10.3390/healthcare13172128

**Published:** 2025-08-27

**Authors:** Francesca Strassoldo di Villanova, Gabriele Morganti, Matteo Vitarelli, Matteo Quarantelli, Bernard Andrieu, Bruno Ruscello, Elvira Padua

**Affiliations:** 1 Department of Human Sciences and Promotion of the Quality of Life, San Raffaele Open University of Rome, 00166 Rome, Italy; gabriele.morganti@uniroma5.it (G.M.); matteo.vitarelli@uniroma5.it (M.V.); matteo.quarantelli@uniroma5.it (M.Q.); bruno.ruscello@uniroma5.it (B.R.); elvira.padua@uniroma5.it (E.P.); 2 Institut des Sciences du Sport-Santé de Paris, Université Paris Cité, F-75015 Paris, France; 3 Research for Athlete and Youth Sport Development (RAYSD) Lab, Centre for Life and Sport Sciences (CLaSS), Faculty of Health, Education and Life Sciences, Birmingham City University, Birmingham B15 3TN, UK; 4 Department of Neurosciences, Biomedicine and Movement, University of Verona, 37134 Verona, Italy; 5 Department of Industrial Engineering, Faculty of Engineering, “Tor Vergata” University, 00133 Rome, Italy; 6 LUISS SportLab, 00197 Rome, Italy

**Keywords:** health, behaviors, physical activity, nutrition, Italian people

## Abstract

**Background:** A health-promoting lifestyle is a concept that involves sociodemographic factors interacting with health-promoting lifestyle behaviors (HPLBs), such as exercise and nutrition, to promote health and wellbeing. Given the persistent socioeconomic disparities all over Italy, health interventions and outcomes may be less effective. Accordingly, this cross-sectional study examines the relationship among sociodemographics, HPLBs, and wellbeing in Italy, aiming to inform updated health campaigns. **Methods:** A Google-Form survey of twelve sociodemographic items and two validated questionnaires on HPLBs (HPLP II, twenty-six items) and wellbeing (WHO-5) was conducted. Three hundred two participants, aged 18 to 70, were recruited in Italy via social media. Descriptive and inferential statistics were performed. Statistical significance was set at *p* < 0.05, considering effect sizes. **Results:** Women reported higher health responsibilities (*p* < 0.05) and lower BMIs than men (*p* < 0.001). An improved financial status was associated with the healthy cut-off points of the WHO-5, HPLP II total score, and spiritual growth and interpersonal relationship subscales (*p* < 0.001), the latter correlating also with university education (*p* < 0.05). Better nutrition was noted in older adults, those with chronic disease, and people from Northern Italy (*p* < 0.05). Residing with minors and adults related to health responsibility (*p* < 0.05). A significant correlation (*p* < 0.001) was found between following HPLBs and wellbeing and among all the HPLBs. **Conclusions:** This study underscores the interplay among sociodemographic factors, HPLBs, and wellbeing among Italian adults. The findings advocate for education-based, inclusive health promotion strategies tailored to specific macroregions, age classes, and sexes in Italy, intending to enhance overall health and wellbeing across the country.

## 1. Introduction

Lifestyle is a multidimensional concept encompassing personal behaviors and sociodemographic characteristics (e.g., sex, age, education, and employment) that influence wellbeing and health outcomes (i.e., risks of morbidity and mortality), as reviewed by Brivio et al. [1]. It involves three dimensions: internal (i.e., values and attitudes), external (i.e., socioeconomic conditions, such as housing, financial status, and employment), and temporal (i.e., behavioral evolution over time). These dimensions should be studied and considered when designing sustainable and targeted interventions to promote disease prevention, higher wellbeing, and a health-promoting lifestyle. The last results from the interaction between the above-mentioned sociodemographic characteristics and the practice of specific behaviors, called health-promoting lifestyle behaviors (HPLBs) (e.g., physical activity, balanced nutrition, stress management, health responsibility, interpersonal relationships, and spiritual growth), are indicated by Savarese et al. [2].

This framework relates to the socioecological model conceptualized by McLeroy et al. [3], an application of Bronfenbrenner’s model [4] in health promotion, stating that health behaviors, herein referred to as HPLBs, are not shaped only by personal choices. Individuals influence and are influenced by their social and physical environments over time. This model considers (i) intrapersonal factors, such as the knowledge they may not have of the risks and the effects of different behaviors and their control over environmental influences, behaving the same way as peer pressure, as seen in smoking; (ii) interpersonal networks, including family, work group, and friends, that can influence the development of some good or bad HPLBs, and being or not being supportive, as in the case of parents who suffer from some form of addiction (e.g., food, drugs, or alcohol) or have active friends and colleagues; (iii) institutional and organizational settings, because many people spend the majority of their lives in schools, universities, and work settings; (iv) relationships among interpersonal and organizational settings, forming local communities; and (v) public policies and laws to protect the health of the community.

Health outcomes and a health-promoting lifestyle are increasingly shaped by the so-called “social determinants of health” (SDHs). These include non-medical factors that influence daily life, such as economic stability, education, access to and quality of healthcare, neighborhood and built environment, and community context. As external factors, they significantly impact the adoption and maintenance of HPLBs by affecting access to resources like healthy food, infrastructure for physical activity, and opportunities for social and spiritual engagement. These determinants also contribute to health inequalities both within and between countries [5]. Therefore, effective health promotion must involve multilevel strategies that recognize and address the complex environments where HPLBs develop, rather than focusing solely on individual changes.

HPLBs are associated with a lower risk of chronic diseases and improved health outcomes, as confirmed by recent studies. For instance, Marino et al. [6] highlighted how diets high in saturated fats and ultra-processed foods, especially when combined with physical inactivity, alcohol consumption, and smoking, are strongly associated with higher risks of cancers, such as breast, colorectal, and prostate. Conversely, healthy dietary patterns, like the Mediterranean diet (i.e., rich in fruits, vegetables, whole grains, legumes, nuts, and olive oil, moderate amounts of fish, and a small amount of wine) along with regular physical activity, sufficient sleep, and abstention from tobacco and alcohol abuse, have been associated with cognitive decline reduction and a lower risk of dementia in older adults [7] and increased longevity even among genetically vulnerable individuals [8].

Sociodemographic variables have been shown to influence the adoption of HPLBs. For instance, Moscatelli et al. [9] found that Italian university students living alone in the south of Italy are less likely to maintain HPLBs. In line with this, Carballo-Fazanes et al. [10] observed that family support and positive school experiences boosted Spanish university students’ physical activity levels, while sedentarism correlated with longer study hours, screen time, and psychological distress. Moreover, Müller et al. [11] reported that German male university students were more engaged in physical activity and strength training, while females tended to prepare healthier meals but may experience more stress-related eating. Higher academic levels were associated with better nutrition and sleep and lower tobacco use. Furthermore, in a study by Flesia et al. [12], high school males from the north of Italy were shown to adopt several risky behaviors (e.g., smoking, alcohol, and/or substance abuse). Shifting to work environments, another study by Christodoulou et al. [13] noted positive links between wellbeing and productivity and Mediterranean diet adherence, particularly among highly educated women working in Greece and Cyprus.

For the reasons above, although the benefits of HPLBs are well documented, (i) individual, (ii) environmental, and (iii) interventional barriers could reduce adherence and compliance. In their recent review, Deslippe et al. [14] described those barriers: (i) low motivation, resistance to change, health limitations (e.g., fatigue and illness), and unrealistic expectations (e.g., rapid weight loss or stress reduction); (ii) lack of social support, social norms and stigma, unchangeable environmental factors (like bad weather), and limited community infrastructure (e.g., lack of sidewalks, cycle lanes, and safe spaces); and (iii) inconvenient diet or training schedules, distant healthcare locations, a one-size-fits-all approach lacking cultural and personal tailoring, lack of health counseling, and reliance on a health professional to maintain a new HPLB. On the other hand, they underscored some facilitators: (i) positive attitude to behavioral change (e.g., desire for knowledge and a positive mindset), health concerns motivating that change (e.g., prevention of chronic disease), and perceived psycho-physical improvements reinforcing adherence (e.g., weight loss and increased vitality); (ii) practical, emotional, and motivational social support from family, peers, and professionals, as well as accessible infrastructures (e.g., parks, gyms, and bike paths and fresh, high-quality foods) that enable a health-promoting lifestyle; and (iii) user-centric health intervention design, including proximity, inclusivity, flexibility, follow-up, personalization, goal setting, and guidance.

Sociodemographic disparities significantly influence wellbeing and the adoption of HPBLs across various regions of a country, such as Italy, which is traditionally divided into three macroregions (i.e., the “northern” macroregion = Emilia–Romagna, Friuli Venezia Giulia, Liguria, Lombardia, Piemonte, Trentino Alto Adige, Valle d’Aosta, and Veneto; the “central” macroregion = Lazio, Marche, Toscana, and Umbria; and the “southern and island” macroregion = Abruzzo, Basilicata, Calabria, Campania, Molise, Puglia, Sardegna, and Sicilia). Additionally, Italian residents are not evenly distributed, with approximately 46% living in the north, 20% in the center, and 34% in the south and islands [15]. These divisions are more than just geographical because they also reflect distinct historical (north vs. south since Italian unity in 1861), cultural, social, and economic traits, even in the same region [16,17]. They still affect disease prevention and mortality rates, complicating the generalization of health survey results and campaigns. In line with this, Petrelli et al. [18] reported that lower educational attainment and residency in the south seemed to be associated with a higher risk of avoidable mortality (i.e., preventable and treatable diseases). Given Italy’s decentralized, bottom-up health promotion strategy, Battel-Kirk et al. [19] found that regional inequalities may persist due to differences in institutional quality, economic development, and healthcare availability across regions, as noted by Abatemarco et al. [20]. They underlined that southern areas often suffer from underfunded systems and a shortage of healthcare professionals. However, even if resource imbalances are also evident in parts of the north, when institutions operate effectively and adhere to national healthcare standards, socioeconomic factors could have less impact on health [21]. Finally, residential satisfaction, considered as the pleasure derived from living in a place according to one’s needs, expectations, and outcomes (i.e., satisfaction with dwelling in a neighborhood, the house, and the architecture), is an essential component of wellbeing in Italy, as shown by Miola et al. [22]. They observed that in Northern Italy, people who are satisfied with their environment experience greater wellbeing.

Accordingly, this study aimed to explore the complex and constantly changing relationships among sociodemographic factors, HPLBs, and subjective wellbeing among Italian adults. We hypothesized that sociodemographic factors would be significantly associated with differences in both HPLB practice and wellbeing, due to the previously mentioned sociodemographic disparities all over the country. Finally, this observational research seeks to provide updated insights, supporting new health promotion studies and strategies in Italy.

## 2. Materials and Methods

### 2.1. Study Design and Ethical Considerations

This cross-sectional quantitative study was conducted by the Forum Sport Center Laboratory in the Department of Human Sciences and the Promotion of Quality of Life at the San Raffaele Open University of Rome according to ethical guidelines for research involving human participants (Helsinki Declaration, 1975 and subsequent amendments) and data protection laws, including the GDPR and Italian Legislative Decree no. 196/2003, as amended by Legislative Decree no. 101/2018. The project was reviewed and approved by the Lazio 5 Ethics and Research Committee on Human Beings (protocol no. 9798). It consisted of an online exploratory national survey released via social networks and online interaction platforms (Instagram, Facebook, and WhatsApp) from December 2024 to the end of April 2025. After reading and agreeing to the informed consent and privacy policy, participants were required to complete a self-reported questionnaire remotely via Google Forms. Finally, they received a free e-book about HPLBs and how to improve their habits, as a reward for their reply.

### 2.2. Sample Size Calculation

The participants were recruited among Italian adults via a Google-Form survey of twelve sociodemographic items and two validated questionnaires (the Health-Promoting Lifestyle Profile II and the WHO-5 Wellbeing Index) from December 2024 to the end of April 2025.

According to standard sampling theory [23,24], a sample of 302 participants yields a margin of error of approximately ±5.6% at a 95% confidence level, which can be considered as acceptable in the case of exploratory analyses with an heterogenous and stratified population for age, sex, and geographical area, using self-reported and validated questionnaires [25,26,27]. Furthermore, future studies supported by institutions could increase the sample size to reduce this margin.

### 2.3. Participants

The study included individuals residing all over Italy (i.e., north = Emilia–Romagna, Friuli Venezia Giulia, Liguria, Lombardia, Piemonte, Trentino Alto Adige, Valle d’Aosta, and Veneto; center = Lazio, Marche, Toscana, and Umbria; and south and islands = Abruzzo, Basilicata, Calabria, Campania, Molise, Puglia, Sardegna, and Sicilia), both male and female, between 18 and 70 years of age. Individuals under 18 were not considered for inclusion due to ethical concerns about obtaining informed consent from minors. Furthermore, since the study was conducted through the administration of an online questionnaire, individuals over the age of 70 were excluded, as they may have limited access to the Internet and, therefore, may lack the means or ability to complete the questionnaire under investigation. The participants were recruited through convenience and snowball sampling [28,29] via social networks and online interaction platforms (Instagram, Facebook, and WhatsApp). To encourage participation, participants were provided with a free e-book on HPLB changes, immediately after completing the survey (attached to the Supplementary Materials as File S1).

### 2.4. Measurements

At first, participants answered 12 sociodemographic questions about age; sex; weight; height; municipality, province, and macroregion of residence; education; employment; housing; financial status; and suffering or not from a chronic disease. This first section was based on and adapted from the previous literature [30,31], pilot-tested for clarity, and validated on a small sample of volunteers before data collection. Specifically, twenty people (ten males and ten females) were asked to complete the survey to eliminate ambiguities, assess the time required to fill out the questionnaire, and ensure the Google Form functioned correctly. The survey was well-presented and clear, and they found that the average time needed was 10 min.

To assess HPLBs, the Health-Promoting Lifestyle Profile II (HPLP II) questionnaire [32] was employed in the Italian validated version of 26 items [2]. It evaluates the frequency of engagement in HPLBs across five subscales: health responsibility (HRESP), physical activity (PHACT), nutrition (NUTRI), spiritual growth (SPGRO), and interpersonal relationships (INTRE). Each item is rated on a four-point Likert scale ranging from 1 (“never”) to 4 (“routinely”), with higher scores indicating greater adoption of HPLBs.

Subjective psychological wellbeing was assessed using the validated WHO-5 Wellbeing Index, a widely used tool developed by the World Health Organization [33]. It comprises five positive-affirmation questions, reflecting the presence of wellbeing during the previous two weeks. Answers followed a six-point Likert scale (from 0 = “at no time” to 5 = “all of the time”), and a higher raw score (0–25) denotes greater wellbeing. A cut-off raw score of below 13 shows reduced wellbeing and potential depressive symptoms. The survey is attached to the Supplementary Materials as File S2.

### 2.5. Data Preparation

1. Standardization: Continuous variables (HPLP II total score, WHO-5 total score, NUTR, PHACT, SPGRO, INTRE, HRESP, BMI, and age) were standardized to z-scores to allow for comparison on a common scale. This transformation was crucial for interpreting the relative importance of predictors in the regression analysis;
2. Creation of Dummy Variables: The categorical variables (sociodemographics) were converted to dummy variables. This encoding allows for the inclusion of categorical data in the regression model, with one category (the reference category) being excluded to avoid multicollinearity.

### 2.6. Data Analysis

Statistical analyses were conducted using Jamovi (version 2.5.7) and IBM-SPSS 27 software. Descriptive analyses, such as frequencies, percentages, means, and standard deviations, were performed to describe the sample, its stratifications (age, sex, and macroregion), and other sociodemographic variables. Mean scores and standard deviations were also computed for the total scores of the WHO-5 index and the HPLP II, along with their subscales.

After Shapiro–Wilk normality assessment, statistical significance was set at *p* < 0.05 for all the tests. Independent-sample Student’s *t*-tests and Mann–Whitney tests explored “sex” and “chronic disease” differences in mean scores of HPLP II subscales, the total score, and the WHO-5 index. One-way ANOVA and Kruskal–Wallis tests assessed differences across other sociodemographic categories. Significant results were followed by post hoc comparisons (i.e., Bonferroni correction and the Dwass–Steel–Critchlow–Fligner procedure) to determine the direction of the observed association.

The magnitude of the effect size was interpreted as follows: (i) Cohen’s D (for independent-sample Student’s *t*-tests and Bonferroni correction) values of <0.2 indicate a very small effect, 0.2 < 0.4 a small effect, 0.5 < 0.8 a medium effect, and ≥0.8 a large effect [34]; (ii) Rank biserial correlation (r) (for the Mann–Whitney test) values of <0.3 indicate a small effect, 0.3 < 0.5 a medium effect, and ≥0.5 a large effect [35]; (iii) omega-squared (ω^2^) values of <0.02 indicated a very small effect, 0.02 < 0.12 indicated a small effect, 0.13 < 0.25 indicated a medium effect, and ≥0.26 indicated a large effect [36]; (iv) epsilon-squared (ε^2^) (for the Kruskal–Wallis test) values of ≤0.01 indicated a negligible effect, 0.01 < 0.04 a small effect, 0.04 < 0.16 a moderate effect, 0.16 < 0.36 relatively strong, 0.36 < 0.64 strong, and ≥0.64 very strong [37,38]. Pearson and Spearman correlation coefficients were calculated to assess the association between overall HPLP II scores (and subscales) and WHO-5 scores, along with age and BMI. Their values were interpreted assuming 0.1 < −0.3 indicated negligible and weak correlations, −0.3 < −0.5 a low positive (negative) correlation, −0.5 < −0.7 a moderate positive (negative) correlation, −0.7 < −0.9 a high positive (negative) correlation, and −0.9 < −1 a very high positive (negative) correlation [39].

The chi-squared test for independence (χ^2^) assessed the association between sociodemographics and WHO-5 index cut-off categories. The effect sizes (Cramer’s V) and odds ratios (ORs) were also calculated to reveal the magnitude and direction of an existing relationship for significant chi-squared outputs.

Cramer’s V was interpreted as follows: 0.05 < 0.09 indicated a weak effect size, 0.10 < 0.14 indicated a moderate effect size, 0.15 < 0.24 indicated a strong effect size, and ≥0.25 indicated a very strong effect size [40]. The ORs and 95% CIs were used to compare sociodemographic variables for their likelihood of developing a healthy WHO-5 cut-off point. CIs including 1 (i.e., 95% CI 0.90–1.10) marked no association.

Three models of multiple linear regression analysis were conducted to examine the relationships between each of the dependent variables (the HPLP II total score and WHO-5 total score) and the independent variables (sociodemographics), as well as between the HPLP II total score and its subscales. Among the predictors, the standardized regression coefficients (β) were used to estimate each variable’s relative contribution to the explained variance. The weights were calculated as a percentage of the total sum of the absolute standardized s.

## 3. Results

### 3.1. Descriptive Statistics of the Sample

**Figure 1:**
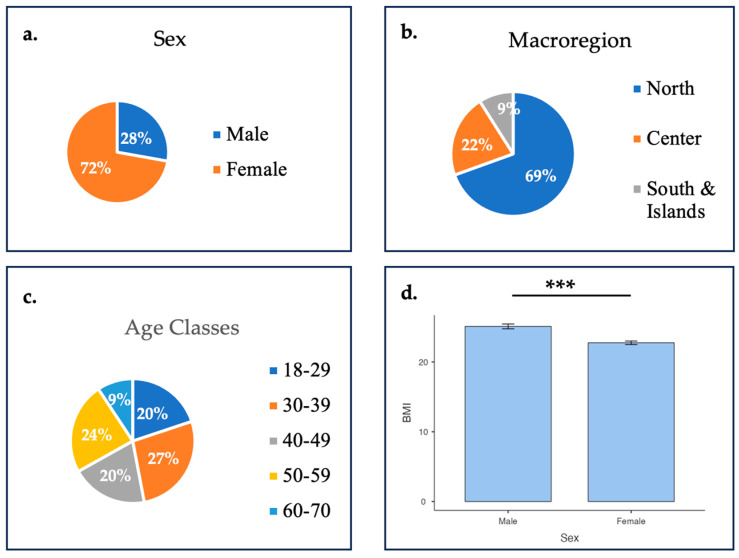
Sample distributions by (**a**) sex, (**b**) macroregion, (**c**) age classes, and (**d**) BMI difference among sexes. *Note:* Mann–Whitney result: *** *p* < 0.001.

The final sample included 302 participants (mean age = 42.2 ± 12 years), with 72.2% female (n = 218; mean age = 42.6 ± 12.3 years) and 27.8% male (n = 84; mean age = 41.2 ± 13 years). Most respondents were from Northern Italy (69.5%), followed by Central (21.5%) and Southern Italy (8.9%). The largest age classes were 18–29 (19.9%), 30–39 (27.1%), 40–49 (19.9%), and 50–59 (23.8%), with only 9.3% in the 60–70 range (Figure 1).

Approximately 68% held a university degree, 29.8% had a high school diploma, and 2.3% had completed middle school. Most participants were employed (85.1%), followed by students (8.9%), unemployed individuals (2.6%), and retirees (3.3%) (Table 1). The mean BMI was 25.1 ± 3.16 kg/m^2^ for men and 22.8 ± 3.74 kg/m^2^ for women, showing consistent trends across age classes and regions, with women having a significantly lower BMI (*p* < 0.001) (Figure 1).

### 3.2. Mean HPLP II Scores, Subscales, and Sociodemographic Differences

According to the Shapiro–Wilk test, the distribution of the total score for HPLP II was normal (*p* > 0.05), whereas its subscales were non-normally distributed (*p* < 0.001). The mean scores for the HPLP II subscales were as follows (Table 2): NUTRI (M = 2.57 ± 0.62), SPGRO (M = 2.87 ± 0.58), PHACT (M = 2.37 ± 0.70), INTRE (M = 2.92 ± 0.48), and HRESP (M = 2.39 ± 0.65). The overall HPLP II score was 69.10 ± 11.3 out of 104, indicating adequate engagement in HPLBs across the sample.

Table 3 summarizes the differences in sociodemographic groups according to HPLP II scores. Independent-sample Student’s *t*-tests and Mann–Whitney tests indicated a significant difference between the *sexes* in the HRESP subscale (*p* < 0.01, small effect), with women scoring higher on average. Nonetheless, other tendencies toward higher scores were observed in men’s PHACT (*p* = 0.06, small effect) and SPGRO (*p* = 0.10, small effect). The same tests were conducted to compare individuals with and without a *chronic disease*. The NUTR subscale (*p* = 0.01, small effect) demonstrated higher mean scores among individuals with a chronic disease.

ANOVA showed a significant result for *Macroregion* with the total HPLP II score (*p* = 0.01, small effect), though post hoc tests found no differences. In contrast, NUTR differences were significant (*p* < 0.01, small-to-moderate effect), with the north group scoring higher than the center (*p* < 0.05) and south groups (*p* < 0.05).

Regarding *Age Classes*, NUTR displayed a significant result (*p* < 0.01, moderate effect), particularly between both 18–29 and 60–70 age classes (*p* < 0.01) and 40–49 and 60–70 (*p* < 0.05), with the latter scoring higher.

*Education* groups seemed to differ in HPLP II scores (*p* < 0.05, small effect), but post hoc analysis did not support this. By contrast, INTRE showed a significant difference (*p* < 0.01, small effect), with the University group scoring higher than the Middle School group (*p* < 0.05).

*Employment* groups did not show any statistically significant difference.

*Housing* showed differences for SPGRO (*p* = 0.01, small effect) and HRESP (*p* < 0.05, small effect), though post hoc tests confirm only the latter, with “living with u18 and other adults” scoring higher than “living alone” (*p* < 0.05) and “living with other adults” (*p* < 0.05).

*Financial Status* groups differed significantly in the HPLP II total score (*p* < 0.001, small effect). Those with a worsened financial status had lower scores compared to those with an unchanged (*p* < 0.05) or an improved status (*p* < 0.001). Unchanged status also scored lower than improved (*p* < 0.05). SPGRO (*p* < 0.001, moderate effect) and INTRE (*p* < 0.001, moderate effect) followed similar patterns.

However, it is important to note that although the statistical significance of most differences is recognized, the practical implications of minor effects may be limited.

### 3.3. Mean WHO-5 Index Score and Cut-Off Point Variations by Sociodemographics

According to the Shapiro–Wilk test, the distribution of the total score for the WHO-5 was not normal (*p* < 0.05). The mean WHO-5 wellbeing index total score was 16.10 ± 3.63 out of 25 (Table 2). Notably, 78.5% of the participants surpassed the WHO-5 healthy cut-off point of 13, indicating a satisfactory level of perceived wellbeing among the sample. A chi-squared test assessed the association between the WHO-5 cut-off point of 13 and sociodemographics. Financial status was the only variable with a significant relationship (*p* < 0.01, strong effect), as shown in Table 4. Additionally, it was expected that only 9% of the people in the sample with a worsened financial status would report lower wellbeing (<13), rather than 18.2%.

### 3.4. Correlations Among Health-Promoting Lifestyle, Wellbeing, Age, and BMI

Correlation matrices identified some correlations, as presented in Table 5. The HPLP II and the WHO-5 total scores demonstrated a positive correlation (*p* < 0.001, weak). Age did not show a significant correlation with the HPLP II total score but exhibited a very weak positive correlation with the WHO-5 total score (*p* < 0.05). BMI showed no significant correlation with the WHO-5 or the HPLP II total score. When examining correlations between the WHO-5 total score and HPLP II subscales, positive but very weak correlations were observed with NUTR (*p* < 0.05), PHACT (*p* < 0.01), and INTRE (*p* < 0.01), and a low positive correlation was observed with SPGRO (*p* < 0.001). Weak-to-low positive correlations were also identified among all the HPLP II subscale scores (*p* < 0.001), except for SPGRO and INTRE, which showed moderate positive correlations.

### 3.5. Regression Analyses of Wellbeing and Health Behaviors

Three multiple linear regressions were used to determine the main predictors of wellbeing (WHO-5) and the adoption of HPLBs (HPLP II total score). The first model (Table 6) examined sociodemographic (entered as dummy variables) and HPLBs (entered as z-scores) as predictors of wellbeing, and was statistically significant (F(13, 288) = 2.55, *p* = 0.002), accounting for 10.3% of the variance in the outcome (adjusted R^2^ = 0.063). The most influential predictor was Financial_status_1 (β = −0.26, *p* < 0.001), contributing 35.2% of the explained variance. Financial _status_2 (β = −0.18, *p* = 0.008) accounted for an additional 24.1%, and Housing_4 (β = 0.13, *p* = 0.026) explained 17.6%. Age (β = 0.10) and BMI (β = −0.08) had smaller, non-significant effects, contributing 12.9% and 10.2% of the total standardized impact, respectively. These findings suggest that financial difficulties are strongly associated with lower engagement in health-promoting behaviors, while favorable housing conditions and age show positive associations. In contrast, higher BMIs are negatively related to health-promoting lifestyles. The second model (Table 7) examined the influences of the same sociodemographic variables on the adoption of HPLBs (HPLP II total score) and was not statistically significant (F(16, 285) = 1.37, *p* = 0.154), explaining approximately 7.2% of the variance in wellbeing. Among the predictors, Chronic_disease_2 (β = 0.073, 11.0%) and Employment_4 (β = 0.072, 10.9%) showed the strongest positive associations with WHO-5 scores. Conversely, Employment_2 (β = −0.064, 9.7%) and Housing_1 (β = −0.056, 8.4%) were negatively associated with wellbeing. Although the model’s explanatory power was limited, these findings suggest that categorical life conditions, such as employment type, chronic illness status, and housing, may contribute to understanding variations in subjective wellbeing. Finally, a third (Table 8) and highly significant model (F(5, 296) = 1604.38, *p* < 0.001) investigated the relationships between the five subscales of the HPLP II and its total score, explaining 96.4% of the variance. All the subscales were significant predictors, with NUTR (β = 0.339) and PHACT (β = 0.319) emerging as the strongest contributors.

## 4. Discussion

In examining the sample, this discussion highlights the potential implications of the sample’s distribution among sex (a higher number of women respondents), macroregion (the south is underrepresented), and age groups (older people are less likely to participate in online surveys). Subsequently, the practice of HPLBs and wellbeing confirms correlations, discussing associations with sociodemographic factors: (i) higher HRESP in women; (ii) better NUTR in the north and among older age groups and people with chronic diseases; (iii) higher education fosters INTRE; (iv) households with minors and other adults increase HRESP; (v) not having to overcome financial problems leads to healthier wellbeing, more SPGRO, and better INTRE. Finally, the practical implications derived from our findings suggest new public health policies and interventions in Italy, encouraging future research and addressing the study’s limitations.

### 4.1. Sample Distribution

The respondents were predominantly women (72.2%), which deviates from the actual population distribution in Italy on 1 January 2025 (51%) [15,16,17,18,19,20,21,22,23,24,25,26,27,28,29,30,31,32,33,34,35,36,37,38,39,40,41]. While the data confirm a slightly higher number of female residents compared to male residents, the disproportionate representation in our sample likely reflects the higher survey response rates typically observed among women [42]. This phenomenon may indicate a greater propensity for self-awareness and interest in health-related self-assessment and responsibility among women, as discussed in the next paragraph. The distribution of respondents across macroregions also showed discrepancies compared to the actual Italian distribution (north: 69.5% vs. 47%; center: 21.5% vs. 20%; south and islands: 8.9% vs. 33%). This uneven representation aligns with observations by Bussola et al. [43], who noted that survey response rates can be influenced by factors such as the survey method (online, as in our case), sociodemographic characteristics (with higher education levels correlating with increased online survey participation), and the region of residence. The underrepresentation of southern respondents in online surveys may reflect regional differences in internet access, digital engagement, and health education. The age classes in our sample were generally well represented, except for individuals aged 60–70, who constituted a smaller proportion. This finding is consistent with ISTAT data [44], indicating lower digital proficiency among older adults in Italy, which may have limited their participation in our online survey.

### 4.2. HPLB Practice Across Sociodemographics and Wellbeing

Our initial hypothesis posited that sociodemographic factors in Italy would impact HPLB practice and wellbeing. Accordingly, we observed a significant difference between sex groups in HRESP, suggesting that women may exhibit greater health-conscious behaviors than men. It correlates with previous findings about the exhibition among men of more health risk behaviors, like substance abuse or seeking health information online instead of consulting a professional [12]. While this is not statistically significant, we also noted tendencies toward sex differences in the WHO-5 total score, PHACT, and SPGRO, warranting further exploration in future studies. This outcome aligns with those of other studies, indicating existing gender-specific patterns in HPLBs [11,12,13,14,15,16,17,18,19,20,21,22,23,24,25,26,27,28,29,30,31,32,33,34,35,36,37,38,39,40,41,42,43,44,45]. Finally, the mean value of the BMI in adult males was significantly higher than that in females, as in Mistura et al.’s recent Italian survey [46].

Despite the responders’ regional distribution, significant differences, with a small-to-moderate effect, were found across Italy for NUTR. Specifically, individuals from the north demonstrated higher engagement in healthy nutrition compared to those from the center, south, and islands. These findings may correspond with the existing literature that emphasizes regional inequalities in Italy. Notable factors include production inefficiencies, insufficient supply of healthcare providers, and disparities in health spending capacity. These issues affect access to professional health guidance (e.g., consulting a dietitian) and the availability of healthy foods, which correlate with a higher prevalence of overweight individuals in the southern regions [15,17,46]. NUTR also reported higher values in people suffering from chronic diseases and across older ages, underlining the roles of the diet in assisting pharmacological therapies and the aging process [47,48].

Education level influenced the INTRE, suggesting that higher education may foster stronger social connections and support networks, as observed. Educational settings play a pivotal role in enhancing students’ relational competence, contributing significantly to the development of interpersonal relationships [49].

Living with minors and other adults appears to correlate more with the need to seek guidance from healthcare professionals than when living alone, with other adults, or only with kids. Living with children often entails additional caregiving responsibilities, which, when shared with other adults (e.g., partners or grandparents), may contribute to a more complex family-related psychosocial burden [50]. This can result in more frequent consultations with healthcare professionals.

A worsened financial status negatively influences the healthy wellbeing cut-off point, along with HPLBs’ practice, especially SPGRO and INTRE. This highlights the crucial role of economic factors as barriers or facilitators in shaping health, wellbeing, sociality, and spirituality [14]. The resulting SPGRO from statements, such as “I feel content and at ease with myself” or “I feel I am changing and growing in a positive way”, along with the INTRE from “spend time with close friends”, indicates that individuals may find it challenging to engage in social activities or maintain their trust in the life process following a negative change in financial status.

The correlation between the HPLP II and WHO-5 total scores, even if small, confirms the already stated connection between the two variables: Individuals following HPLBs tend to report higher levels of wellbeing and vice versa. The significant linear regression models showed that factors such as the macroregion of residence, education, housing, and financial status influenced the adoption of health behaviors, though these factors explained only a small portion of the variability. Moreover, both NUTR and PHACT were confirmed to significantly impact HPLBs. In addition, all the findings above underscore the complex interplay among sociodemographic factors, HPLBs, and wellbeing in Italy. For this reason, institutions must periodically investigate these variables and their associations to adjust health promotion strategies.

### 4.3. Practical Implications

This study adds to the existing body of literature by offering updated insights into the practice of HPLBs and the wellbeing of the Italian population. The findings underscore the necessity of reconsidering sociodemographic factors in the design and execution of new public health interventions and policies in Italy.

Key recommendations include (Figure S1 in the Supplementary Materials):

- Prioritizing the center, the south, and the islands, where HPLBs are less prevalent, by increasing access to healthcare services, encouraging community involvement, and promoting health professional education;
- Boosting health education in middle and high schools, as well as free-access workplace welfare programs across Italy, to foster long-term improvements in HPLBs, particularly among individuals with lower levels of formal education;
- Promoting inclusive and meaningful free activities all over Italy, such as community sports, mindfulness, and volunteer programs, given that PHACT, SPGRO, and INTRE significantly predict wellbeing;
- Encouraging participation in wellness initiatives through economic and environmental policies, including free annual access to personalized physical activity or nutritional plans;
- Building more functional exercise spaces accessible to all ages and increasing the availability of green areas and natural environments in urban settings;
- Enhancing digital literacy among older adults or designing tailored health interventions still using non-digital methods;
- These strategies would reduce health disparities, mortality rates, and hospitalization costs.

### 4.4. Limits

This study has some limitations. First, the use of convenience and snowball sampling methods, combined with an online self-reported survey, may have introduced selection bias (e.g., exclusion of older adults and people from the center, the south, the islands, and/or those from lower socioeconomic backgrounds), impacting the representativeness of the Italian population. Furthermore, relying on self-assessment might have been influenced by social desirability and recall bias. The margin of error is approximately ±5.6% at a 95% confidence level, which could affect the generalizability of the findings to the broader Italian population. Finally, the cross-sectional design limits the ability to infer causal relationships between sociodemographic factors and HPLBs and perceived wellbeing.

### 4.5. Perspectives

Future studies should aim to recruit more balanced and larger samples across sex, age classes, and macroregional distribution, potentially involving institutional partnerships (e.g., hospitals, schools, universities, and companies) and mixed recruitment (e.g., phone interviews, community outreach, and paper surveys) and data collection strategies, thus obtaining a broader representation of the population, including elderly age classes, males, and people from the center, the south, and the islands of Italy. Longitudinal designs could help to explore causal relationships and temporal dynamics between wellbeing and lifestyle behaviors. Additionally, qualitative approaches, such as interviews, may provide deeper insights into individual and sociocultural barriers to or facilitators of health promotion. Lastly, intervention studies could evaluate the effectiveness of tailored strategies in improving HPLBs and wellbeing in specific subgroups, such as managing menstrual cycle issues in women, reducing men’s health risk behaviors, or investigating perceptions and needs of people living in different Italian macroregions, with a focus on the center, the south, and the islands.

## 5. Conclusions

This study confirms the correlation between the practice of HPLBs and the feeling of wellbeing among Italian adults. Factors such as age, education, financial status, and housing are important contributors. Especially, not having to overcome financial problems, a higher education, an older age stage, and being a woman seem to be associated more with HPLBs and a healthy wellbeing. To better interpret the results of geographical comparisons, further studies, supported by regional institutions, may be necessary to ensure a sample representing the south and the islands coherently. It will be important to address regional differences as well.

Summing up, these results highlight the need for updating inclusive, region-specific, and education-focused public health strategies to improve a health-promoting lifestyle and overall wellbeing in Italy. The present survey can be a useful tool for institutions to evaluate HPLBs and wellbeing over time in Italy.

## Figures and Tables

**Table 1:** Sociodemographic frequencies.

Sociodemographic Factor	Percentage (N)
**Sex**	
Male	27.8% (84)
Female	72.2% (218)
**Age classes**	
18–29	19.9% (60)
30–39	27.1% (82)
40–49	19.9% (60)
50–59	23.8% (72)
60–70	9.3% (28)
**Macroregions**	
North	69.5% (210)
Center	21.5% (65)
South and Islands	8.9% (27)
**Education**	
Middle school	2.3% (7)
High school	29.8% (90)
University	68% (205)
**Employment**	
Student	8.9% (27)
Unemployed	2.6% (8)
Employed	85.1% (257)
Retired	3.3% (10)
**Housing**	
Living with u18 and other adults	8% (25)
Living with u18	9% (28)
Living with other adults	58% (174)
Living alone	25% (75)
**Financial status**	
Worsened	9% (28)
Unchanged	69% (207)
Improved	22% (67)
**Chronic disease**	
Yes	14% (41)
No	86% (261)

**Table 2:** Descriptives of HPLP II and WHO-5 index scores.

Questionnaire Results	Total Score Mean ± SD (% ON MAX Scoring)
WHO-5 total score	16 ± 3.63 (64.4%)
HPLP II total score	69.1 ± 11.3 (66%)
HPLP II subscales	
NUTR	2.57 ± 0.62
SPGRO	2.87 ± 0.58
PHACT	2.37 ± 0.70
INTRE	2.92 ± 0.48
HRESP	2.39 ± 0.65

**Table 3:** Differences between sociodemographic factors and scores of HPLP II and its subscales.

Variable	HPLP II Total Score	NUTR	PHACT	INTRE	HRESP	SPGRO
	Mean ± SD	Mean ± SD	Mean± SD	Mean ± SD	Mean ± SD	Mean ± SD
**Sex**						
Male	69.5 ± 11.00	2.51 ± 0.60	2.49 ± 0.67	2.87 ± 0.48	2.23 ± 0.65	2.98 ± 0.57
Female	68.9 ± 11.4	2.59 ± 0.62	2.32 ± 0.71	2.93 ± 0.48	2.45 ± 0.64	2.83 ± 0.58
*p-value* ^1^	*p* = 0.72	*p* = 0.26	*p* = 0.056	*p* = 0.25	*p* = 0.002 **	*p* = 0.098
*ES*	*d* = 0.05	*r* = 0.08	*r* = −0.14	*r* = 0.08	*r* = 0.22	*r* = −0.12
**Age Classes**						
18–29	67.5 ± 12.0	2.39 ± 0.65 ^a^	2.37 ± 0.77	3.00 ± 0.50	2.31 ± 0.67	2.79 ± 0.62
30–39	70.3 ± 11.30	2.60 ± 0.64 ^ab^	2.32 ± 0.72	2.97 ± 0.45	2.47 ± 0.64	2.93 ± 0.56
40–49	69.0 ± 10.70	2.49 ± 0.65 ^a^	2.36 ± 0.65	2.94 ± 0.42	2.35 ± 0.71	2.98 ± 0.53
50–59	68.4 ± 11.60	2.64 ± 0.53 ^ab^	2.38 ± 0.69	2.80 ± 0.50	2.36 ± 0.61	2.85 ± 0.59
60–70	70.6 ± 9.92	2.87 ± 0.50 ^b^	2.48 ± 0.60	2.84 ± 0.52	2.50 ± 0.57	2.70 ± 0.56
*p-value* ^2^	*p* = 0.583	*p* = 0.008 **	*p* = 0.875	*p* = 0.111	*p* = 0.387	*p* = 0.118
*ES*	*ω*^2^ = −0.004	*ε*^2^ = 0.05 ^#^	*ε*^2^ = 0.004	*ε*^2^ = 0.02	*ε*^2^ = 0.01	*ε*^2^ = 0.02
**Macroregions**						
North	70.3 ± 11.00	2.64 ± 0.62 ^a^	2.41 ± 0.70	2.96 ± 0.46	2.42 ± 0.63	2.89 ± 0.57
Center	66.6 ± 11.90	2.45 ± 0.56 ^b^	2.26 ± 0.68	2.82 ± 0.55	2.36 ± 0.71	2.78 ± 0.62
South and Islands	65.6 ± 10.70	2.30 ± 0.60 ^b^	2.26 ± 0.73	2.79 ± 0.46	2.22 ± 0.65	2.91 ± 0.55
*p-value* ^2^	*p* = 0.017 ^N.S.^	*p* = 0.005 **	*p* = 0.192	*p* = 0.063	*p* = 0.354	*p* = 0.205
*ES*	*ω*^2^ = 0.02	*ε*^2^ = 0.04	*ε*^2^ = 0.01	*ε*^2^ = 0.02	*ε*^2^ = 0.01	*ε*^2^ = 0.01
**Education**						
Middle school	61.1 ± 5.93	2.17 ± 0.45	1.91 ± 0.65	2.54 ± 0.30 ^a^	2.03 ± 0.49	2.74 ± 0.55
High school	67.3 ± 10.80	2.54 ± 0.61	2.38 ± 0.67	2.83 ± 0.47 ^ab^	2.28 ± 0.60	2.79 ± 0.53
University	70.1 ± 11.40	2.60 ± 0.62	2.38 ± 0.71	2.97 ± 0.48 ^b^	2.45 ± 0.66	2.91 ± 0.60
*p-value* ^2^	*p* = 0.022 ^N.S.^	*p* = 0.179	*p* = 0.234	***p*** = 0.006 **	*p* = 0.025 N.S.	*p* = 0.123
*ES*	*ω*^2^ = 0.02	*ε*^2^ = 0.01	*ε*^2^ = 0.01	*ε*^2^ = 0.03	*ε*^2^ = 0.02	*ε*^2^ = 0.01
**Employment**						
Student	70.0 ± 12.30	2.43 ± 0.67	2.50 ± 0.72	3.09 ± 0.55	2.35 ± 0.73	2.92 ± 0.67
Unemployed	67.3 ± 13.10	2.52 ± 0.89	2.50 ± 0.71	2.75 ± 0.32	2.52 ± 0.79	2.60 ± 0.59
Employed	69.1 ± 11.20	2.58 ± 0.61	2.35 ± 0.71	2.92 ± 0.47	2.40 ± 0.64	2.89 ± 0.56
Retired	67.1 ± 8.75	2.80 ± 0.39	2.45 ± 0.49	2.64 ± 0.39	2.29 ± 0.54	2.56 ± 0.73
*p-value* ^2^	*p* = 0.876	*p* = 0.352	*p* = 0.718	*p* = 0.044 ^N.S.^	*p* = 0.750	*p* = 0.150
*ES*	*ω*^2^ = −0.01	*ε*^2^ = 0.01	*ε*^2^ = 0.004	*ε*^2^ = 0.03	*ε*^2^ = 0.004	*ε*^2^ = 0.02
**Housing**						
Living with u18 and other adults	73.5 ± 10.30	2.74 ± 0.51	2.42 ± 0.76	3.06 ± 0.43	2.73 ± 0.65 ^a^	3.09 ± 0.45
Living with u18	70.3 ± 12.20	2.46 ± 0.52	2.38 ± 0.63	3.09 ± 0.47	2.46 ± 0.72 ^ab^	3.10 ± 0.62
Living with other adults	68.4 ± 11.00	2.56 ± 0.62	2.33 ± 0.69	2.88 ± 0.48	2.35 ± 0.64 ^b^	2.84 ± 0.57
Living alone	68.8 ± 11.60	2.58 ± 0.66	2.44 ± 0.74	2.89 ± 0.48	2.34 ± 0.61 ^c^	2.79 ± 0.60
*p-value* ^2^	*p* = 0.177	*p* = 0.256	*p* = 0.665	*p* = 0.057	*p* = 0.038 *	*p* = 0.016 ^N.S.^
*ES*	*ω*^2^ = 0.006	*ε*^2^ = 0.01	*ε*^2^ = 0.005	*ε*^2^ = 0.02	*ε*^2^ = 0.03	*ε*^2^ = 0.03
**Financial Status**						
Worsened	62.9 ± 10.90 ^a^	2.47 ± 0.64	2.23 ± 0.66	2.56 ± 0.45 ^a^	2.34 ± 0.65	2.38 ± 0.51 ^a^
Unchanged	68.8 ± 10.80 ^b^	2.57 ± 0.60	2.35 ± 0.68	2.89 ± 0.46 ^b^	2.41 ± 0.65	2.86 ± 0.55 ^b^
Improved	72.5 ± 11.70 ^c^	2.60 ± 0.65	2.48 ± 0.78	3.16 ± 0.43 ^c^	2.37 ± 0.64	3.10 ± 0.53 ^c^
*p-value* ^2^	*p* < 0.001 ***	*p* = 0.567	*p* = 0.192	***p*** < 0.001 ***	*p* = 0.711	***p*** < 0.001 ***
*ES*	*ω*^2^ = 0.04	*ε*^2^ = 0.003	*ε*^2^ = 0.01	*ε*^2^ = 0.10 ^#^	*ε*^2^ = 0.002	*ε*^2^ = 0.09 ^#^
**Chronic Disease**						
Yes	70.2 ± 9.90	2.79 ± 0.55	2.36 ± 0.68	2.99 ± 0.47	2.50 ± 0.677	2.84 ± 0.60
No	68.9 ± 11.50	2.54 ± 0.62	2.37 ± 0.70	2.91 ± 0.48	2.37 ± 0.642	2.88 ± 0.58
*p-value* ^1^	*p* = 0.498	***p*** = 0.014 *	*p* = 0.943	*p* = 0.172	*p* = 0.309	*p* = 0.562
*ES*	*d* = −0.11	*r* = 0.24	*r* = 0.007	*r* = 0.13	*r* = 0.09822	*r* = −0.05

*Notes:* WHO-5, World Health Organization 5 wellbeing index; HPLP II health-promoting lifestyle profile II 26 items; SD, standard deviation; ES, effect size. ^1^ Differences are based on independent-sample Student’s *t*-test and Mann–Whitney test. ^2^ Differences are based on parametric and non-parametric ANOVA; *** p < 0.001; ** *p* < 0.01; * *p* < 0.05. ^a,b,c^ Results with different superscript letters in variables with more than two categories indicate significant differences by post hoc tests, while ^N.S.^ indicates there is no significant difference by post hoc tests; ^#^ moderate–large effect sizes in significant results.

**Table 4:** Chi-squared test for the WHO-5 cut-off points across the financial status.

WHO-5 Cut-Off Point	Worsened (Expected)	Unchanged (Expected)	Improved (Expected)	*χ* ^2^	V	*p*-VALUE	EFFECT
**>13**	16 (21.9)	163 (162.4)	58 (52.6)				
%	6.8 (9.2)	68.8 (68.5)	24.5 (22.2)				
**<13**	12 (6)	44 (44.6)	9 (14.4)				
%	18.5 (9%)	67.7 (68.6)	13.8 (22.1)				
				10.1	0.18	**0.006**	Strong

*Notes: χ*^2^ results, chi-square test; V, Cramer’s V; significant results asre in bold, ***p* < 0.05.**

**Table 5:** Correlation matrix.

Variables	WHO-5 TS	HPLP II TS	PHACT	INTRE	HRESP	SPGRO
	**Ρ**	**r**	**ρ**	**ρ**	**ρ**	**ρ**
**AGE**	0.116 *	0.001				
**BMI**	0.017	−0.090				
**HPLP II TOTAL SCORE**	0.246 ***	-				
**NUTR**	0.133 *	-	0.487 ***	0.254 ***	0.359 ***	0.313 ***
**PHACT**	0.164 **	-	-	0.275 ***	0.228 ***	0.429 ***
**INTRE**	0.146 **	-		-	0.379 ***	0.616 ***
**HRESP**	0.034	-			-	0.310 ***
**SPGRO**	0.360 ***					-

*Notes*: WHO-5 TS, WHO-5 wellbeing index total score; HPLP II TS, health-promoting lifestyle profile II 26-item total score; r and ρ, correlation coefficients; *p < 0.05; ** *p* < 0.01; *** *p* < 0.001.

**Table 6:** Multiple linear regression coefficients for predictors of the HPLP II total score.

Coefficients ^a^										
Model		Unstandardized Coefficients		Standardized Coefficient	t	Sig.	95.0% Confidence Interval for B		Collinearity Statistics	
		B	Std. Error	Beta			Lower Bound	Upper Bound	Tolerance	VIF
1	(Constant)	76.832	2.610		29.439	0.000	71.695	81.969		
	Macroregion_of_residence_2	−4.255	1.572	−0.156	−2.707	0.007	−7.349	−1.161	0.915	1.092
	Macroregion_of_residence_3	−5.014	2.292	−0.127	−2.188	0.030	−9.526	−0.503	0.893	1.119
	Education_1	−7.137	4.199	−0.096	−1.699	0.090	−15.403	1.129	0.957	1.045
	Education_2	−3.179	1.450	−0.129	−2.192	0.029	−6.033	−0.324	0.869	1.151
	Employment_1	3.903	2.549	0.099	1.531	0.127	−1.115	8.921	0.722	1.385
	Employment_2	2.124	4.007	0.030	0.530	0.596	−5.763	10.011	0.923	1.084
	Employment_4	−2.137	3.751	−0.034	−0.570	0.569	−9.521	5.247	0.848	1.179
	Chronic_desease_2	−2.136	1.874	−0.065	−1.140	0.255	−5.826	1.553	0.927	1.079
	Housing_1	0.745	1.528	0.029	0.488	0.626	−2.262	3.752	0.877	1.140
	Housing_3	2.936	2.308	0.076	1.272	0.204	−1.608	7.480	0.852	1.173
	Housing_4	5.111	2.356	0.125	2.169	0.031	0.473	9.749	0.906	1.103
	Financial_status_1	−9.546	2.531	−0.246	−3.772	0.000	−14.527	−4.565	0.709	1.410
	Financial_status_2	−3.923	1.593	−0.162	−2.463	0.014	−7.058	−0.788	0.698	1.432
	Sex_2	−1.474	1.528	−0.059	−0.965	0.335	−4.481	1.533	0.815	1.226
	Z-score: Age	1.116	0.786	0.099	1.420	0.157	−0.431	2.664	0.620	1.612
	Z-score: BMI	−1.239	0.678	−0.110	−1.829	0.068	−2.573	0.094	0.835	1.197

^a^ Dependent variable: HPLP II total score.

**Table 7:** Multiple linear regression coefficients for predictors of the WHO-5 wellbeing index total score.

Coefficients ^a^										
Model		Unstandardized Coefficients		Standardized Coefficient	t	Sig.	95.0% Confidence Interval for B		Collinearity Statistics	
		B	Std. Error	Beta			Lower Bound	Upper Bound	Tolerance	VIF
1	(Constant)	16.811	0.874		19.231	0.000	15.090	18.532		
	Sex_2	−0.812	0.512	−0.100	−1.588	0.113	−1.820	0.195	0.815	1.226
	Macroregion_of_residence_2	0.460	0.527	0.052	0.874	0.383	−0.576	1.497	0.915	1.092
	Macroregion_of_residence_3	1.266	0.768	0.100	1.649	0.100	−0.245	2.777	0.893	1.119
	Education_1	−0.977	1.407	−0.041	−0.695	0.488	−3.746	1.791	0.957	1.045
	Education_2	−0.643	0.486	−0.081	−1.323	0.187	−1.599	0.313	0.869	1.151
	Employment_1	−0.458	0.854	−0.036	−0.537	0.592	−2.139	1.223	0.722	1.385
	Employment_2	−1.451	1.342	−0.064	−1.081	0.281	−4.092	1.191	0.923	1.084
	Employment_4	1.463	1.256	0.072	1.164	0.245	−1.010	3.936	0.848	1.179
	Chronic_disease_2	0.771	0.628	0.073	1.229	0.220	−0.464	2.007	0.927	1.079
	Housing_1	−0.470	0.512	−0.056	−0.918	0.359	−1.477	0.537	0.877	1.140
	Housing_3	−0.315	0.773	−0.025	−0.407	0.684	−1.837	1.207	0.852	1.173
	Housing_4	−0.068	0.789	−0.005	−0.086	0.931	−1.621	1.485	0.906	1.103
	Financial_status_1	−1.608	0.848	−0.129	−1.897	0.059	−3.277	0.060	0.709	1.410
	Financial_status_2	−0.697	0.534	−0.089	−1.307	0.192	−1.748	0.353	0.698	1.432
	Z-score: Age	0.506	0.263	0.139	1.922	0.056	−0.012	1.024	0.620	1.612
	Z-score: BMI	−0.043	0.227	−0.012	−0.189	0.850	−0.490	0.404	0.835	1.197

^a^ Dependent variable: WHO-5 total score.

**Table 8:** Multiple linear regression coefficients for the prediction of the HPLP II total score based on standardized subscale scores.

Coefficients ^a^										
Model		Unstandardized Coefficients		Standardized Coefficient	t	Sig.	95,0% Confidence Interval for B		Collinearity Statistics	
		B	Std. Error	Beta			Lower Bound	Upper Bound	Tolerance	VIF
1	(Constant)	69.086	0.123		560.955	0.000	68.844	69.328		
	Z-score: Nutrition	3.815	0.146	0.339	26.041	0.000	3.526	4.103	0.709	1.410
	Z-score: Spiritual Growth	3.176	0.167	0.282	18.994	0.000	2.847	3.505	0.544	1.837
	Z-score: Physical Activity	3.588	0.148	0.319	24.259	0.000	3.297	3.879	0.696	1.438
	Z-score: Interpersonal Relationships	2.605	0.163	0.232	15.977	0.000	2.284	2.926	0.573	1.747
	Z-score: Health Responsibility	2.492	0.141	0.222	17.691	0.000	2.215	2.770	0.767	1.304

^a^ Dependent variable: HPLP II total score.

## Data Availability

The data associated with this paper are available from the corresponding author upon request.

## Supplementary Materials

The following supporting information can be downloaded at https://www.mdpi.com/article/10.3390/healthcare13172128/s1: File S1: Free e-book; File S2: Survey; Figure S1: Key recommendations.

## Abbreviations

The following abbreviations are used in this manuscript:

HPLBs	Health-Promoting Lifestyle Behaviors
HPLP II	Health-Promoting Lifestyle Profile II
WHO-5	WHO-5 Wellbeing Index
NUTR	Nutrition
PHACT	Physical Activity
HRESP	Health Responsibility
SPGRO	Spiritual Growth
INTRE	Interpersonal Relationships
