# Supplementary material for: Exploring the Interplay Among a Health-Promoting Lifestyle, Wellbeing, and Sociodemographic Characteristics in Italy: A Cross-Sectional Study
Source: Healthcare (Basel). 2025 Aug 27;13(17):2128. doi: 10.3390/healthcare13172128 (PMC12428026; doi:10.3390/healthcare13172128)
Supplement: Supplementary file 1 [file healthcare-13-02128-s001.zip › File S1 - Free E-book.pdf]

Università San Raffaele
Roma

# Lifestyle, Habits, and Quality of Life

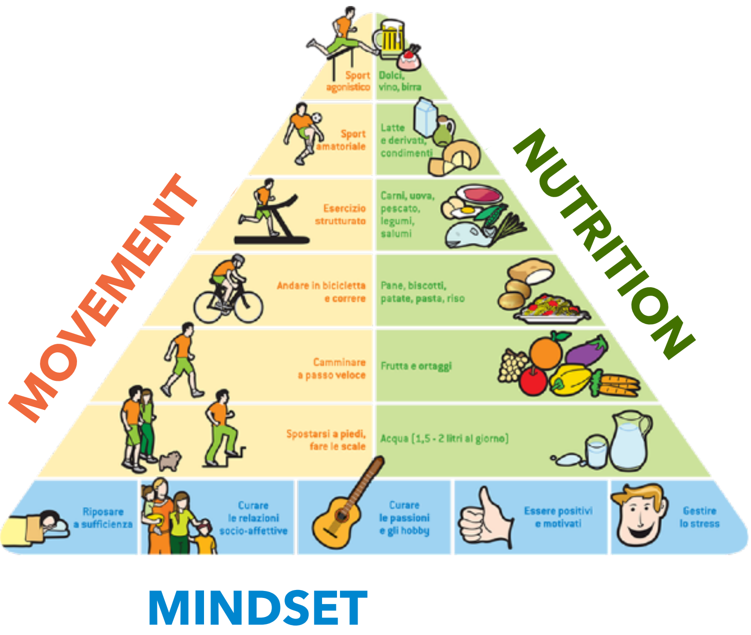


**Research Team:** Dott. Gabriele Morganti, Dott. Matteo Vitarelli, Dott.ssa Francesca Strassoldo di Villanova

# *INDICE*

**Changing Lifestyle** **3**

*What does it mean?*

**Changing Lifestyle** **5**

*5 Useful Steps*

**Food – Macronutrients** **7**

*What are their functions?*

**Movement** **11**

*BENEFITS*

# WHAT DOES IT MEAN?

1. Lifestyle includes the way we live and our daily habits (nutrition, physical activity, relationships, sleep, hobbies, work, etc.) in a 360° view: it is never static.

2. Nutrition and physical activity are just two pieces of a much bigger puzzle. They both influence and are influenced by other aspects of our life. These are key habits: by improving them, other areas of life also improve like a domino effect.

3. To change lifestyle, a simple diet or workout plan is not enough. These are prescriptions that may not be tailored to you, may lack real meaning, and often bring only temporary results. You are not the active protagonist in choosing the right behaviors for yourself. Guidelines are important, of course, but they must also be "ecological" for your life – that is, respectful of your priorities, preferences, needs, commitments, and relationships.

4. Pleasure is crucial for lifestyle change. We tend to repeat automatic behaviors because they once gave us gratification or still do. Some no longer gratify us, but we keep doing them out of habit. Others may only gratify us in the short term, but they could be replaced with behaviors that are satisfying both immediately and in the long run. Example: Before bed, drinking milk and eating cookies may be pleasant and relaxing, but it can lead to weight gain. Alternatives like taking a hot bath, drinking herbal tea, reading, stretching while listening to music, petting your pet, or massaging with a cream are just as relaxing and enjoyable without negative consequences. In the long term, they are even more rewarding.

# 5 USEFUL STEPS

1. Get the right information about nutrition and exercise, and make it yours: what macronutrients and water are for, how to compose a balanced plate, the benefits of movement, and the importance of pleasure in lifestyle change.

2. Write down what you already do: start from point A (your current habits). Keep a diary of what you eat, your physical activity, hydration, what feels easy or hard, and your emotions. Identify what gives you positive results (keep it) and what bothers you (change it). Clearly define point B, your specific goal for the coming months, using the SMART method (specific, measurable, achievable, realistic, time-bound).

3. Change one behavior at a time, replacing it with something that is enjoyable both now and in the future.

4. Start with simple actions and repeat them, even the ones that seem obvious. If they were truly obvious, you would already be doing them.

5. Be the active protagonist of your choices and make sure they fit your environment (priorities, relationships, places, available time). Enjoy it and make it an essential ritual!

# WHAT ARE THEIR FUNCTIONS?

1. Nutrition education starts with the basics: understanding the functions of foods and macronutrients, which are present in the foods we commonly eat.

## Food Nutrients and Their Functions

| Nutrients | Functions |
|---|---|
| **Carbohydrates** | • Energetic<br>• Plastic (Structural)<br>• Regulatory/Functional<br>• Reserve<br>• Calming<br>• Effects related to dietary fiber |
| **Proteins** | • Plastic/Supportive/Structural<br>• Regulatory/Functional<br>• Energetic<br>• Long-term Satiety |
| **Fats (Lipids)** | • Energetic<br>• Reserve<br>• Structural<br>• Regulatory/Functional<br>• Long-term Satiety<br>• Palatability (Appetizing) |

2. Each food generally contains one predominant macronutrient (greater in grams compared to the others). For example, pasta and flour mainly contain carbohydrates.

3. Use the following "Food–Macronutrient" table to train yourself in mentally categorizing foods by their main macronutrient.

| CARBOHYDRATES | ...of which FIBER | PROTEIN | FATS |
|---|---|---|---|
| Cereals and partly Legumes | Whole Grain Products | Red Meat | Nuts and Derivatives |
| and derivatives | Vegetables | White Meat | Oilseeds |
| Flours and foods containing flour | Fruit | Fish, Mollusks, Crustaceans... | Extra Virgin Olive Oil |
| Bread and Baked Goods | Legumes | Dairy Products and Derivatives (cheeses) | Avocado |
| Pasta | Nuts | Eggs | Vegetable Oils |
| Potatoes | | Legumes and Veg Derivatives | Butter |
| Fruit | | Un-cured Meats | Vegetable Margarine |
| Honey and Sugar | | Cured Meats | Pesto/Spreadable Creams/Ready-made Sauces/Pâté |
| Jams | | | |
| (Dairy Products)... | | | |

4. An ideal plate (for breakfast, lunch, dinner, or snacks) should always include carbohydrates + proteins + fats + fiber.

- Breakfast should be a “smaller meal,” with smaller portions compared to lunch and dinner.

- For snacks, you can mix a carbohydrate with a protein and/or fat source (e.g., fruit + a piece of Parmesan cheese).

## 5. **WATER**

**Functions?**

- maintain blood volume/blood pressure
- functioning of metabolic processes
- transport and dilution of substances in the body
- control of body temperature
- elimination of waste products
- lubrication of cavities
- maintain intestinal regularity
- PERFORMANCE/muscle function

- **Hydration note:** drinking too little water is harmful, just as drinking too much can be poorly absorbed. Track your water intake for a couple of days. If you drink less than 1 liter, increase gradually, one glass per week, drinking small sips during the day. Aim for about 300–400 ml per 10 kg of body weight (slightly less if sedentary, but never below 1.5 L). During intense activity or in hot weather, add 500 ml per hour of activity. Signs of good hydration include light-yellow urine and urinating every 1.5–3 hours. Tap water is fine if you like it, otherwise, alternate with medium- and low-mineral bottled water. Make hydration enjoyable with reusable bottles, flavored teas, juices, smoothies, and seasonal fruits and vegetables.

# BENEFITS

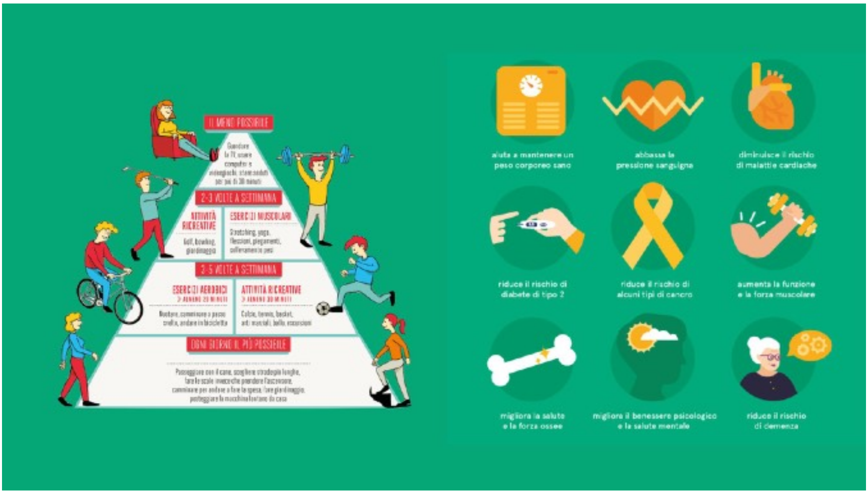


1. Are there aspects of your current life that bother you? Low energy, poor concentration, back pain, tension, excess weight, poor memory, mood swings, anxiety, unstable blood pressure, etc.? These are motivations to include small or longer movement practices that can solve your problems. Exercising just “because it’s good for you” rarely motivates people. But when you feel the real benefits and make it your own, it becomes natural daily practice.

2. Going to the gym twice a week but sitting for 12 hours a day is not better than someone who doesn't go to the gym but moves mainly by walking. The foundation of the movement "pyramid" is daily dynamism, then gradually integrating other activities like sports, cycling, or gym workouts.

3. Changing movement habits, like any lifestyle habit, starts with small, simple, and enjoyable steps that you can repeat consistently until they become automatic and personally rewarding. Start from your current state A (track it), set a clear and concrete point B (your goal), and define small steps to reach it in the long term. Awareness and philosophy are useful, but change truly happens through action – in the body and thanks to the body!

© Copyright 2024 Francesca Strassoldo di Villanova

This work is published and sent directly by the author and the research team via email to the respondents of the survey *"An investigation into the health-promoting lifestyle among Italian individuals and its interaction with sociodemographic variables and personal characteristics."* All rights are exclusively reserved. No part of this book may be reproduced, copied, or shared without the prior consent of the author.

Università San Raffaele
Roma

# Stile di vita, abitudini e qualità di vita

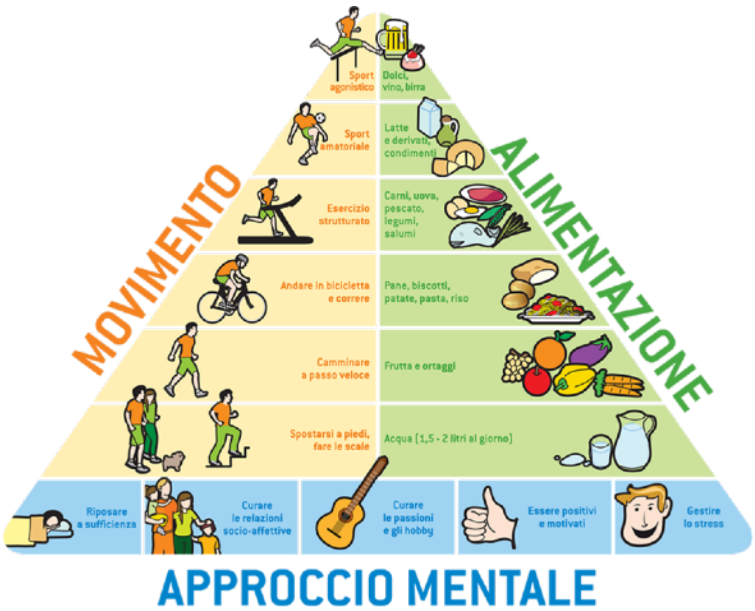


**Team di ricerca:** Dott. Gabriele Morganti, Dott. Matteo Vitarelli, Dott.ssa Francesca Strassoldo di Villanova

*INDICE*

**Cambiare stile di vita** **3**

*CHE COSA SIGNIFICA?*

**Cambiare stile di vita** **5**

*5 PASSI UTILI*

**Alimenti - Macronutrienti** **7**

*MACROS: FUNZIONI?*

**Movimento** **10**

*benefici*

# CHE COSA SIGNIFICA?

1. Lo stile di vita, ingloba il nostro vivere e le nostre abitudini (ALIMENTAZIONE, MOVIMENTO, RELAZIONI, SONNO, HOBBIES, LAVORO…) a 360°: non è mai statico.

2. L'alimentazione e il movimento sono solo un paio di tasselli, in un quadro molto più ampio, ma influenzano e sono influenzati, anche da tutti gli altri pezzetti che compongono il puzzle. Sono abitudini chiave: migliorandole, si migliora a effetto domino anche altri ambiti della nostra vita.

3. Per cambiare lo stile di vita una dieta o una scheda di allenamento non sono sufficienti, perché esegui delle prescrizioni, di cui non sai il motivo e non ne capisci la reale individualizzazione o piacere. Il risultato estetico/di salute spesso è solo momentaneo perché non sei stato/a veramente il protagonista in modo attivo nella scelta dei comportamenti giusti per te: ok le linee guida, certo, ma devono essere soprattutto "ecologici"

per la tua vita e come la vuoi vivere (cioè rispettare le tue priorità, i tuoi gusti, le tue necessità, i tuoi impegni, le tue relazioni ecc).

4. Per cambiare stile di vita è importante il principio del PIACERE! Ripetiamo comportamenti automatici perché in risposta a uno stimolo ci avevano dato o ci danno ancora una gratificazione. Alcuni però ce la davano anni fa, ma ora non ce la danno più seppur non siamo capaci di disinnescarli. Altri ci danno gratificazione solo nell'immediato, e potrebbero essere sostituiti con comportamenti soddisfacenti nell'immediato, ma anche nel lungo periodo. (Esempio: prima di dormire mangio latte e biscotti, è piacevole e mi rilassa ma sto prendendo peso; prima di dormire faccio un bagno caldo/bevo una tisana e leggo un libro/faccio stretching e ascolto musica rilassante/ accarezzo il mio animale/ mi metto la crema e mi faccio un massaggio ecc… è rilassante e piacevole e non mi fa prendere peso = più gratificante di latte e biscotti)

# 5 PASSI UTILI

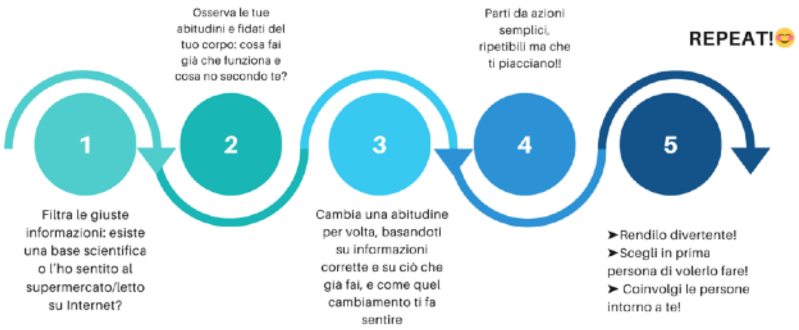


1. Ottieni le giuste informazioni, ad esempio a riguardo della nutrizione e dell'allenamento, e falle tue: a cosa servono i macronutrienti e l'acqua, come comporre un piatto ideale, quali sono i benefici del movimento, quanto è importante il piacere nel cambiamento dello stile di vita e delle nostre abitudini.

2. Annotati cosa fai già: parti dal punto attuale A (scrivi un diario alimentare e del movimento che fai, di come bevi, di ciò che ti viene facile fare e ciò che ti viene difficile, delle tue emozioni). Ciò che senti ti dia un buon risultato in

termini di benessere oggettivo/soggettivo lo tieni, ciò che ti infastidisce invece ti proponi di modificarlo. Definisci anche in modo chiaro (SMART: specifico, misurabile, raggiungibile, realistico e definito nel tempo) il punto B: il tuo obiettivo e cosa vorresti ottenere di concreto tra qualche mese.

3. Cambia un comportamento alla volta e sostituiscilo con un altro più piacevole nell'immediato e nel lungo periodo.

4. Parti dal semplice e ripetilo, anche dalle cose che ti sembrano più banali e scontate, ma non lo sono (perché se lo fossero le staresti già facendo).

5. Sii il protagonista del comportamento scelto e fai in modo che si integri con l'ambiente che ti circonda (priorità, relazioni, luoghi, tempo a disposizione). Divertiti e rendilo un rituale irrinunciabile!

# MACROS: FUNZIONI?

1. Si parte sempre dalle basi dell'Educazione Alimentare, ovvero conoscere le funzioni degli alimenti e dei macronutrienti (che sono contenuti negli alimenti che solitamente mangiamo).

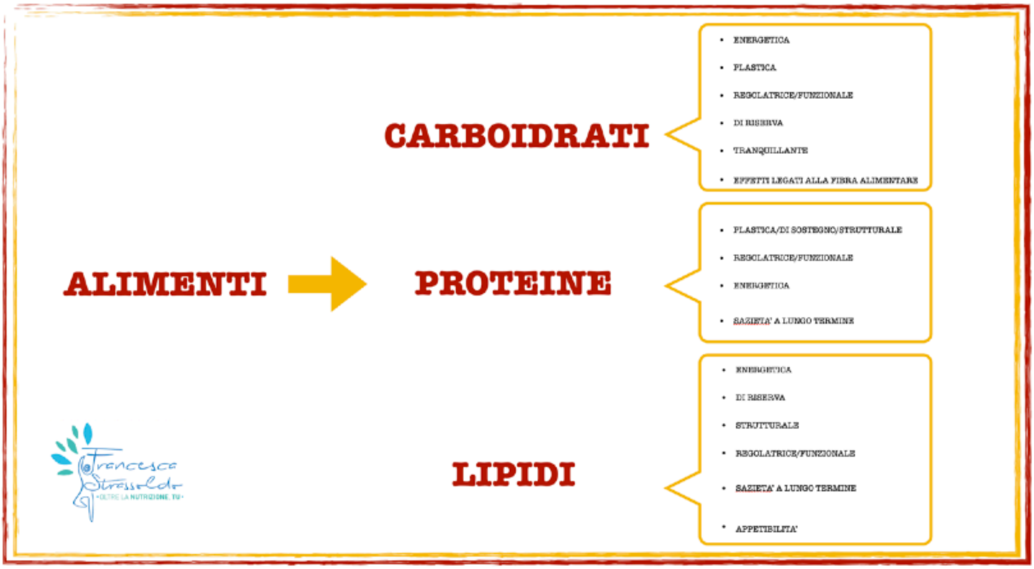


2. Gli alimenti contengono infatti ciascuno un macronutriente preponderante (quantità in grammi maggiore rispetto agli altri macronutrienti contenuti nello

stesso alimento: lo puoi leggere nell'etichetta nutrizionale). Ad es. pasta e farina contengono soprattutto carboidrati.

3. Puoi visionare questa tabella "Alimenti-Macronutrienti" per creare una abitudine mentale alla loro categorizzazione in Macronutrienti di appartenenza.

| CARBOIDRATI | ...di cui FIBRA | PROTEINE | LIPIDI |
|---|---|---|---|
| CEREALI e in parte LEGUMI | PRODOTTI INTEGRALI | CARNE ROSSA | FRUTTA SECCA E DERIVATI |
| e derivati: | VERDURA | CARNE BIANCA | SEMI OLEOSI |
| • FARINE e alimenti contenenti farine | FRUTTA | PESCE, MOLLUSCHI, CROSTACEI... | OLIO EXTRAVERGINE D'OLIVA |
| • PANE e PRODOTTI DA FORNO | LEGUMI | LATTICINI E DERIVATI (formaggi) | AVOCADO |
| • PASTA | FRUTTA SECCA | UOVA | OLI VEGETALI |
| PATATE | | LEGUMI E DERIVATI VEG | BURRO |
| FRUTTA | | SALUMI NON INSACCATI | MARGARINE VEG |
| MIELE E ZUCCHERI | | SALUMI INSACCATI | PESTO/CREME SPALMABILI/SUGHI PRONTI/PATE' |
| MARMELLATE | | | |
| (LATTICINI)... | | | |

4. Il piatto ideale (colazione - pranzo - cena - spuntino ideale) andrebbe sempre composto da alimenti contenenti carboidrati+proteine+lipidi+fibre (a colazione lo renderò "un piccolo pasto" diminuendo le quantità del cibo scelto rispetto a pranzo e cena, mentre negli spuntini se sento l'esigenza di farli potrò mixare una fonte di carboidrati + una fonte di proteine e/o lipidi come ad esempio un frutto come carboidrato + un pezzetto di parmigiano come fonte proteica.

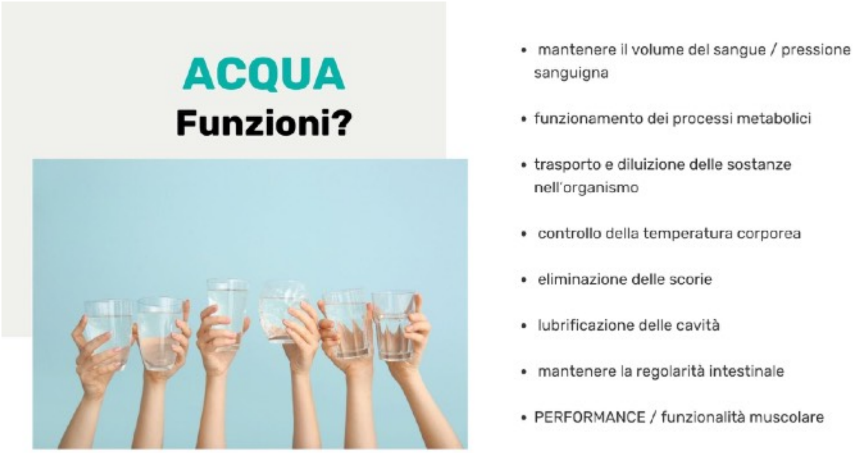


**NB:** poca acqua fa male come bere tanta acqua mal assorbita. Traccia quanta ne bevi per un paio di giorni e se ne bevi meno di un litro aumenta ogni settimana di un bicchiere, bevendo a piccoli sorsi durante il giorno, fino a coprire più o meno il fabbisogno di 300-400 ml per 10kg di peso corporeo (se non si fa molto movimento va bene assumerne anche un pochino meno ma mai sotto 1,5l). In attività fisiche e in stagioni in cui la sudorazione è copiosa, 500ml all'ora, sempre a piccoli sorsi, extra rispetto a quella conteggiata prima. Le urine devono essere gialline e la frequenza di minzione ogni 1,5-2h circa o 3h se si è fuori casa/ufficio. L'acqua del rubinetto è ok se ti piace o mixa medio minerale e oligominerale per la restante. Va resa un'abitudine piacevole: usa borracce termiche carine e comode da trasportare, tisane gustose, centrifugati, e consuma anche sempre frutta/verdura/passati di verdura o minestroni/insalatone in base alla stagione per idratarti anche grazie al cibo.

# BENEFICI

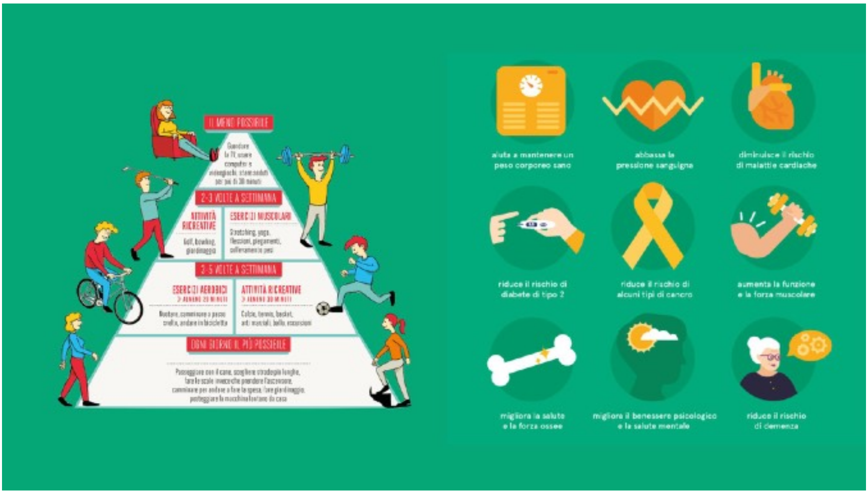


1. Ci sono aspetti della tua vita attuale che ti infastidiscono? Scarsa concentrazione ed energia, mal di schiena, tensioni, peso in eccesso, poca memoria, umore variabile, ansia, pressione instabile (ecc)… Quella è la tua motivazione per dedicarti a piccole o più lunghe pratiche di movimento, che possano risolvere il tuo problema. Fare movimento solo perché fa bene, non ha mai motivato nessuno a farlo. Ma farlo perché lo senti benefico per te, e trovare il tuo modo di viverlo come tale, te lo renderà una pratica quotidiana naturale (siamo fatti per muoverci, a partire dallo spostarci,

conversare, fare attività lavorative o extralavorative: anche quello è fare movimento).

2. Andare in palestra 2 volte alla settimana, ma poi stare seduti 12 ore al giorno non è meglio rispetto a chi non fa palestra, ma si muove prevalentemente a piedi. La base della piramide del movimento richiede dinamicità in generale nella propria vita e poi l'integrazione, via via che diviene naturale per noi, di altre attività, come lo sport, la bicicletta o la palestra.

3. Cambiare abitudini di movimento, come ogni altro cambiamento delle abitudini, parte da abitudini piccole, semplici e piacevoli per te, che possano essere ripetute costantemente nel tempo fino a diventare automatiche e di cui riconosci fortemente il beneficio individuale. Anche in questo caso parti dallo stato A attuale (traccialo): datti un obiettivo B finale, e definisci dei piccoli passi migliorativi da compiere per raggiungerlo a lungo termine in modo chiaro e concreto. La filosofia e la consapevolezza sono utile, ma ci fanno spesso restare nella testa e nel dovrei fare o nella procrastinazione. Il cambiamento avviene anche nel corpo e grazie al corpo, grazie all'azione!

© *Copyright* 2024 Francesca Strassoldo di Villanova

Questa opera è pubblicata ed inviata direttamente dall'autore e dal team di ricerca via mail ai rispondenti alla survey "Un'indagine sullo stile di vita che promuove la salute tra gli individui italiani e la sua interazione con le variabili sociodemografiche e le caratteristiche personali" che detengono ogni diritto della stessa in maniera esclusiva. Nessuna parte di questo libro può essere pertanto riprodotta, copiata o condivisa senza il preventivo assenso dell'autore.
