# Supplementary material for: Exploring the Interplay Among a Health-Promoting Lifestyle, Wellbeing, and Sociodemographic Characteristics in Italy: A Cross-Sectional Study
Source: Healthcare (Basel). 2025 Aug 27;13(17):2128. doi: 10.3390/healthcare13172128 (PMC12428026; doi:10.3390/healthcare13172128)
Supplement: Supplementary file 1 [file healthcare-13-02128-s001.zip › File S2 - SURVEY.pdf]

SURVEY (via Google Form)

SECTION 1 – *SOCIODEMOGRAPHIC VARIABLES AND PERSONAL CHARACTERISTICS*

1. AGE (in numbers):

2. HEIGHT (in centimeters):

3. WEIGHT (in kilograms):

4. GENDER:
- Male
- Female
- Other
- Prefer not to answer

5. CITY/TOWN OF RESIDENCE:

6. PROVINCE OF RESIDENCE:

7. ITALIAN MACROREGION OF RESIDENCE:
- Northern Italy (Emilia-Romagna, Friuli-Venezia Giulia, Liguria, Lombardy, Piedmont, Trentino-Alto Adige, Valle d'Aosta, Veneto)
- Central Italy (Lazio, Marche, Tuscany, Umbria)
- Southern Italy and Islands (Abruzzo, Basilicata, Calabria, Campania, Molise, Apulia, Sardinia, Sicily)

8. EDUCATION LEVEL:
- Middle school diploma
- High school diploma
- University degree

9. EMPLOYMENT STATUS:
- Student
- Employed
- Unemployed
- Retired

10. CHRONIC DISEASE:
- Yes
- No

11. HOUSING:
- I live alone
- I live with other adults
- I live with people under 18
- I live with both adults and people under 18

12. FINANCIAL STATUS (trend in the last 3 months):
- Improved
- Unchanged
- Worsened

SECTION 2 – *LIFESTYLE AND HEALTH PROMOTION*
(*The Health Promoting Lifestyle Profile II – 26 items by Savarese et al., 2018*)

This questionnaire contains a series of statements about your current lifestyle and personal habits. We invite you to answer each statement as accurately as possible and to complete all items. Please indicate how often you engage in each behavior by selecting: N for "Never", S for "Sometimes", O for "Often", or R for "Routinely".

1. Discuss my problems and concerns with people close to me.
2. Choose a diet low in fat, saturated fat, and cholesterol.
3. Report any unusual signs or symptoms to a physician or other health professional.
4. Follow a planned exercise program.
5. Feel I am growing and changing in positive ways.
6. Limit use of sugars and foods containing sugar (sweets).
7. Exercise vigorously for 20 or more minutes at least three times a week (e.g., brisk walking, bicycling, aerobic dancing, and using a stair climber).
8. Believe that my life has purpose.
9. Maintain meaningful and fulfilling relationships with others.
10. Question health professionals to understand their instructions.
11. Take part in light-to-moderate physical activity.
12. Spend time with close friends.
13. Eat 2–4 servings of fruit each day.

14. Take part in leisure-time (recreational) physical activities (e.g., swimming, dancing, and bicycling).
15. Feel content and at peace with myself.
16. Find it easy to show concern, love, and warmth to others.
17. Eat 3–5 servings of vegetables each day.
18. Discuss my health concerns with health professionals.
19. Do stretching exercises at least 3 times per week.
20. Work toward long-term goals in my life.
21. Touch and am touched by the people I care about.
22. Find each day interesting and challenging.
23. Ask for information from health professionals about how to take good care of myself.
24. Am aware of what is important to me in life.
25. Read labels to identify nutrients, fats, and sodium content in packaged food.
26. Reach my target heart rate when exercising.

SECTION 3 – *WHO Well-Being Questionnaire (5 items) (1998 version)*

Please indicate for each of the five statements which is closest to how you have been feeling over the last two weeks (0 = Never; 1 = Sometimes; 2 = Less than half of the time; 3 = More than half of the time; 4 = Most of the time; 5 = Always). Notice that higher numbers mean better well-being. Example. If you have felt cheerful and in good spirits more than half of the time during the last two weeks, select number three.

1. I have felt cheerful and in good spirits.
2. I have felt calm and relaxed.
3. I have felt active and vigorous.
4. I woke up feeling fresh and rested.
5. My daily life has been filled with things that interest me.

🇮🇹

SEZIONE 1 – *VARIABILI SOCIODEMOGRAFICHE E CARATTERISTICHE PERSONALI*

1. ETA' (IN CIFRE):

2. ALTEZZA (IN CENTIMETRI):

3. PESO (IN KILOGRAMMI):

4. SESSO:

• Maschio

• Femmina

• Altro

• Preferisco non rispondere

5. COMUNE DI RESIDENZA:

6. PROVINCIA DI RESIDENZA:

7. MACROREGIONE ITALIANA DI RESIDENZA:

• Nord Italia (Emilia-Romagna, Friuli-Venezia Giulia, Liguria, Lombardia, Piemonte, Trentino-Alto Adige, Valle d'Aosta, Veneto)

• Centro Italia (Lazio, Marche, Toscana, Umbria)

• Sud Italia e Isole (Abruzzo, Basilicata, Calabria, Campania, Molise, Puglia, Sardegna, Sicilia)

8. LIVELLO DI EDUCAZIONE:

- Licenza media
- Diploma di scuola superiore
- Laurea

9. OCCUPAZIONE:

- Studente/ssa
- Occupato/a
- Disoccupato/a
- Pensionato/a

10. MALATTIA CRONICA:

- Sì
- No

11. SITUAZIONE ABITATIVA:

- Vivo da solo/a
- Vivo con altri adulti
- Vivo con under 18
- Vivo con altri adulti e under 18

12. SITUAZIONE FINANZIARIA (andamento negli ultimi 3 mesi):

- Migliorata
- Invariata
- Peggiorata

SEZIONE 2 - *STILE DI VITA E PROMOZIONE DELLA SALUTE (The Health Promoting Lifestyle Profile II 26-items)*

Questo questionario contiene una serie di affermazioni riguardanti il tuo attuale stile di vita ovvero le abitudini personali. Ti invitiamo a rispondere a ciascuna affermazione il più accuratamente possibile; cerca anche di non saltare alcuna voce. Per rispondere indica la frequenza con la quale ti impegni in ogni comportamento segnando: M per "mai", Q per "qualche volta", S per "spesso", oppure D per "di continuo".

1.Discuto i miei problemi e preoccupazioni con persone che mi stanno vicino

2. Scelgo una dieta con pochi lipidi, grassi saturi e colesterolo

3. Riferisco qualunque segno o sintomo a un medico o altro professionista della salute

4. Svolgo esercizio fisico in base ad un programma

5. Sento che sto crescendo e cambiando in modo positivo

6. Limito l'uso di zucchero e cibi contenenti zucchero (dolci)

7. Faccio esercizio fisico vigoroso per 20 o più minuti almeno tre volte a settimana (come correre, andare in bici, aerobica etc.)

8. Ritengo che la mia vita abbia uno scopo

9. Mantengo con gli altri relazioni significative e soddisfacenti

10. Discuto con i professionisti della salute per riuscire a comprendere bene le loro per riuscire a comprendere bene le loro istruzioni

11. Svolgo una attività fisica leggera o moderata (come camminare a passo spedito 30 - 40 minuti almeno 5 volte a settimana)

12. Passo del tempo con i miei amici più cari

13. Mangio 2 - 4 porzioni di frutta ogni giorno

14. Prendo parte ad attività fisiche (ricreative) nel tempo libero (come nuotare, ballare, andare in bicicletta)

15. Mi sento contento ed in pace con me stesso

16. Riesco facilmente a mostrare preoccupazione, amore e affetto agli altri

17. Mangio 3-5 porzioni di verdure ogni giorno

18. Discuto i miei problemi di salute con professionisti della salute

19. Faccio esercizi di allungamento (stretching) almeno 3 volte a settimana

20. Lavoro per obiettivi a lungo termine

21. Tocco e vengo toccato dalle persone che ho a cuore

22. Trovo ogni giorno interessante e stimolante

23. Chiedo informazioni ai professionisti della salute per sapere come prendermi cura di me stesso

24. Sono consapevole di ciò che è importante per me nella vita
importante per me nella vita

25. Leggo le etichette per identificare i nutrienti, i grassi ed il sodio nei cibi

26. Raggiungo la frequenza cardiaca prevista quando faccio esercizio

SEZIONE 3 – *Questionario dell'OMS (in 5 domande) sullo stato di benessere (versione del 1998)*

Per ciascuna delle cinque affermazioni, la preghiamo di indicare la risposta che più si avvicina a come si è sentito/a nelle ultime due settimane. (0= MAI; 1= A VOLTE; 2= MENO DELLA META' DEL TEMPO; 3= PIU' DELLA META' DEL TEMPO; 4= LA MAGGIOR PARTE DEL TEMPO; 5= SEMPRE. I numeri più alti corrispondono a un maggior stato di benessere. Esempio: se nelle ultime due settimane si è sentito/a allegro/a e di buon umore per più della metà del tempo, faccia una crocetta nella casella corrispondente.

1. Mi sono sentito/a allegro/a e di buon umore

2. Mi sono sentito/a calmo/a e rilassato/a

3. Mi sono sentito/a attivo/a ed energico/a

4. Mi sono svegliato/a sentendomi fresco/a e riposato/a

5. La mia vita di tutti i giorni è stata piena di cose che mi interessano
