# Supplementary figures and images for: Exploring the Interplay Among a Health-Promoting Lifestyle, Wellbeing, and Sociodemographic Characteristics in Italy: A Cross-Sectional Study
Source: Healthcare (Basel). 2025 Aug 27;13(17):2128. doi: 10.3390/healthcare13172128 (PMC12428026; doi:10.3390/healthcare13172128)

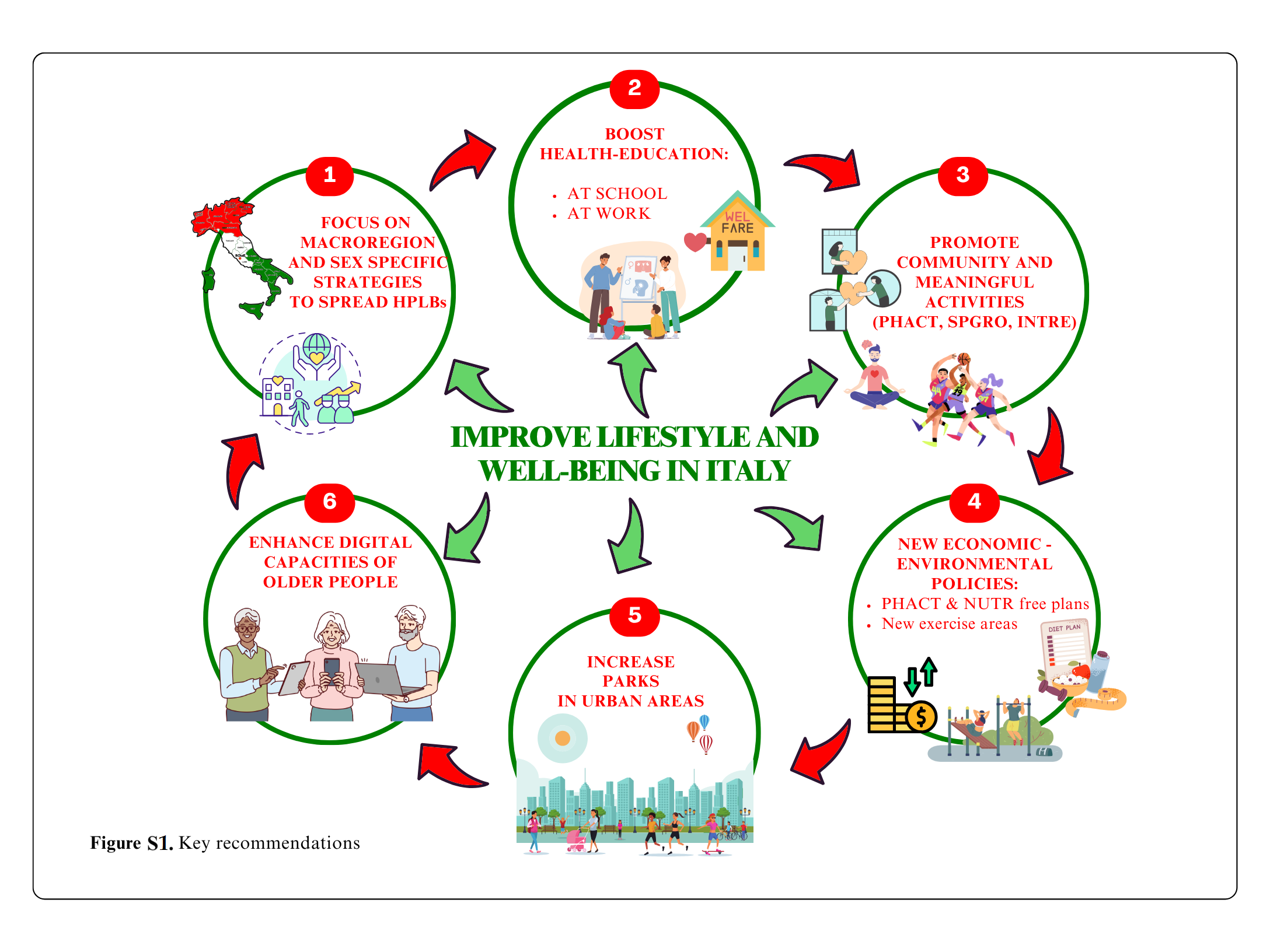

Supplement: Supplementary file 1 [file healthcare-13-02128-s001.zip › Figure S1 Key recommendations.png]
